# A New Medical Journal

**Published:** 1880-06

**Authors:** 


### A NEW MEDICAL JOURNAL.

The first number of the *Arkansas Medical Monthly* has just made its appearance. This is the first medical journal of Arkansas, I think, and makes a good appearance for a beginning; it is filled with interesting and instructive matter. This will doubtless make a new starting point of progress for the medical profession in that growing and rapidly developing State. May it live long and bring forth much fruit.

